# Shift work influences the outcomes of *Chlamydia* infection and pathogenesis

**DOI:** 10.1038/s41598-020-72409-5

**Published:** 2020-09-21

**Authors:** Stephanie R. Lundy, Shakyra Richardson, Anne Ramsey, Debra Ellerson, Yan Fengxia, Sunny Onyeabor, Ward Kirlin, Winston Thompson, Carolyn M. Black, Jason P. DeBruyne, Alec J. Davidson, Lilly C. Immergluck, Uriel Blas-Machado, Francis O. Eko, Joseph U. Igietseme, Qing He, Yusuf O. Omosun

**Affiliations:** 1 Department of Microbiology, Biochemistry & Immunology, Morehouse School of Medicine, 720 Westview Drive, S.W., Atlanta, GA 30310 USA; 2 Department of Neurobiology, Morehouse School of Medicine, Atlanta, GA 30310 USA; 3 Centers for Disease Control & Prevention (CDC), Atlanta, GA 30333 USA; 4 Department of Community Health and Preventive Medicine, Morehouse School of Medicine, Atlanta, GA 30310 USA; 5 Department of Pharmacology, Morehouse School of Medicine, Atlanta, GA 30310 USA; 6 Department of Physiology, Morehouse School of Medicine, Atlanta, GA 30310 USA; 7 Pediatric Clinical & Translational Research Unit, Clinical Research Center, Morehouse School of Medicine, Atlanta, GA 30310 USA; 8 Athens Veterinary Diagnostic Laboratory, Department of Pathology, College of Veterinary Medicine, University of Georgia, Athens, GA 30602 USA

**Keywords:** Microbiology, Bacterial pathogenesis

## Abstract

Shift work, performed by approximately 21 million Americans, is irregular or unusual work schedule hours occurring after 6:00 pm. Shift work has been shown to disrupt circadian rhythms and is associated with several adverse health outcomes and chronic diseases such as cancer, gastrointestinal and psychiatric diseases and disorders. It is unclear if shift work influences the complications associated with certain infectious agents, such as pelvic inflammatory disease, ectopic pregnancy and tubal factor infertility resulting from genital chlamydial infection. We used an Environmental circadian disruption (ECD) model mimicking circadian disruption occurring during shift work, where mice had a 6-h advance in the normal light/dark cycle (LD) every week for a month. Control group mice were housed under normal 12/12 LD cycle. Our hypothesis was that compared to controls, mice that had their circadian rhythms disrupted in this ECD model will have a higher *Chlamydia* load, more pathology and decreased fertility rate following *Chlamydia* infection. Results showed that, compared to controls, mice that had their circadian rhythms disrupted (ECD) had higher *Chlamydia* loads, more tissue alterations or lesions, and lower fertility rate associated with chlamydial infection. Also, infected ECD mice elicited higher proinflammatory cytokines compared to mice under normal 12/12 LD cycle. These results imply that there might be an association between shift work and the increased likelihood of developing more severe disease from *Chlamydia* infection.

## Introduction

There has been an increase in the number of people who work irregular or unusual hours compared to the normal daytime work schedule that occurs between 6:00 am and 6:00 pm^1^. It has been estimated that approximately 21 million Americans, 17% of the United States work force, are considered shift workers^1^. This is due to the increased demand for goods and services in the industrialized world. Shift work causes a disruption in circadian rhythms of people who have these irregular work schedule^2^.

This disruption of the circadian rhythm due to shift work has been associated with adverse health outcomes and disease ranging from chronic conditions such as cancer, cardiovascular, gastrointestinal and psychiatric diseases and disorders^1,3–5^. Shift work has been linked to reproductive disorders ranging from menstrual cycle irregularity, subfecundity, endometriosis, infertility, miscarriage^6^, low birth weight or pre-term delivery and spontaneous abortions^6^. However, the link between shift work and infectious disease progression has not been fully examined, it is thus still unclear if or how shift work increases one’s susceptibility to infectious diseases^7–9^.

Genital chlamydial infection*,* a sexually transmitted infection (STI), caused by the bacteria *Chlamydia trachomatis,* is the most reported bacterial STI in the United States. A high number of *Chlamydia* infection cases in women are unreported because they are asymptomatic^10^. In the female reproductive tract, disease from genital *Chlamydia* infection is manifested in several ways such as pelvic inflammatory disease (PID), Salpingitis (inflammation of the fallopian tubes) and tubal factor infertility^10^. There are varying levels of severity among women who develop complications regardless of how many times they have been exposed or have become reinfected^11,12^. We do not understand why women develop such varying severity in reproductive tract pathology following *Chlamydia* infection.

We had recently reported that the time of day of chlamydial infection was associated with the pathogenesis of *Chalmydia*sis^13^. That study showed a tentative association between disease outcomes of *Chlamydia* infection and circadian rhythms, suggesting that a functional host circadian clock may be necessary for the host to defend itself against *Chalmydia*^13^. In the current report, we disrupted mouse circadian rhythms by changing the light/dark (LD) cycle to simulate shift work^14–17^ and hypothesized that mice subjected to a weekly 6-h advanced shift in their normal light dark cycle and infected with *Chlamydia muridarum* (*C. muridarum)* will show increased infectivity, dysregulated immune profile and increased pathology. In addition, we also compared infection in the early rest period and active period in these mice with disrupted circadian rhythms.

## Results

### Effects of ECD on Chlamydia infectivity

**Figure 1:**
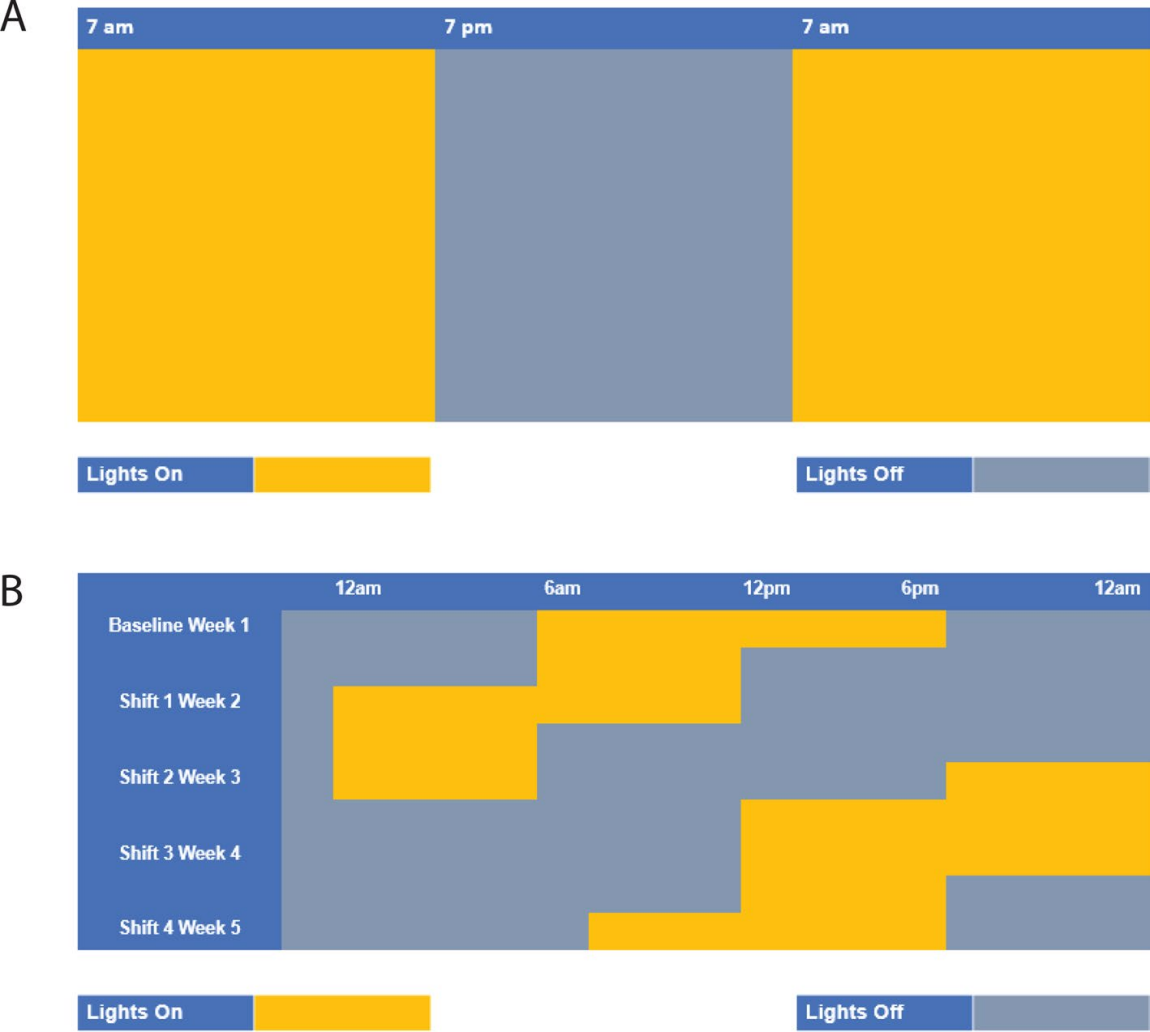
Light cycle conditions. (**A**) Normal light: dark cycle (Control)—mice were housed in cages under normal light: dark cycle (LD) conditions of 12 h light on and 12 h lights out. (**B**) Environmental circadian disruption (ECD) model—mice were housed in cages and received 6-h advance in light cycle every week for 4 weeks.

**Figure 2:**
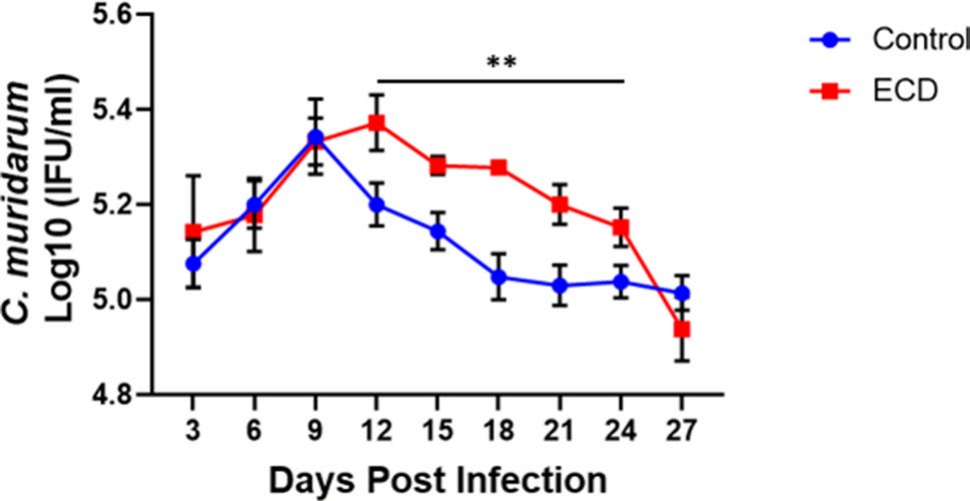
Effect of ECD on *Chlamydia* infectivity. ECD and control mice (n = 12 per group) were infected with *C. muridarum* at ZT3. Data was analyzed using two-way repeat measure ANOVA and Tukey post hoc test (**p < 0.01).

Mice were housed either under normal LD conditions (control) or a 6-h advance in their light cycle every week for 4 weeks (Environmental circadian disruption, ECD, model) (Fig. 1). These mice were then infected with 1 × 10^6^ IFUs of *Chlamydia muridarum (C. muridarum)* at ZT3, the early rest period. ECD Mice had moderate but significant (p < 0.01) *Chlamydia* loads between days 12 and 24 compared to control mice (Fig. 2). This result implies that disruption of circadian rhythm in mice affects chlamydial infectivity.

### Effect of ECD on female reproductive tract pathology after Chlamydia infection

**Figure 3:**
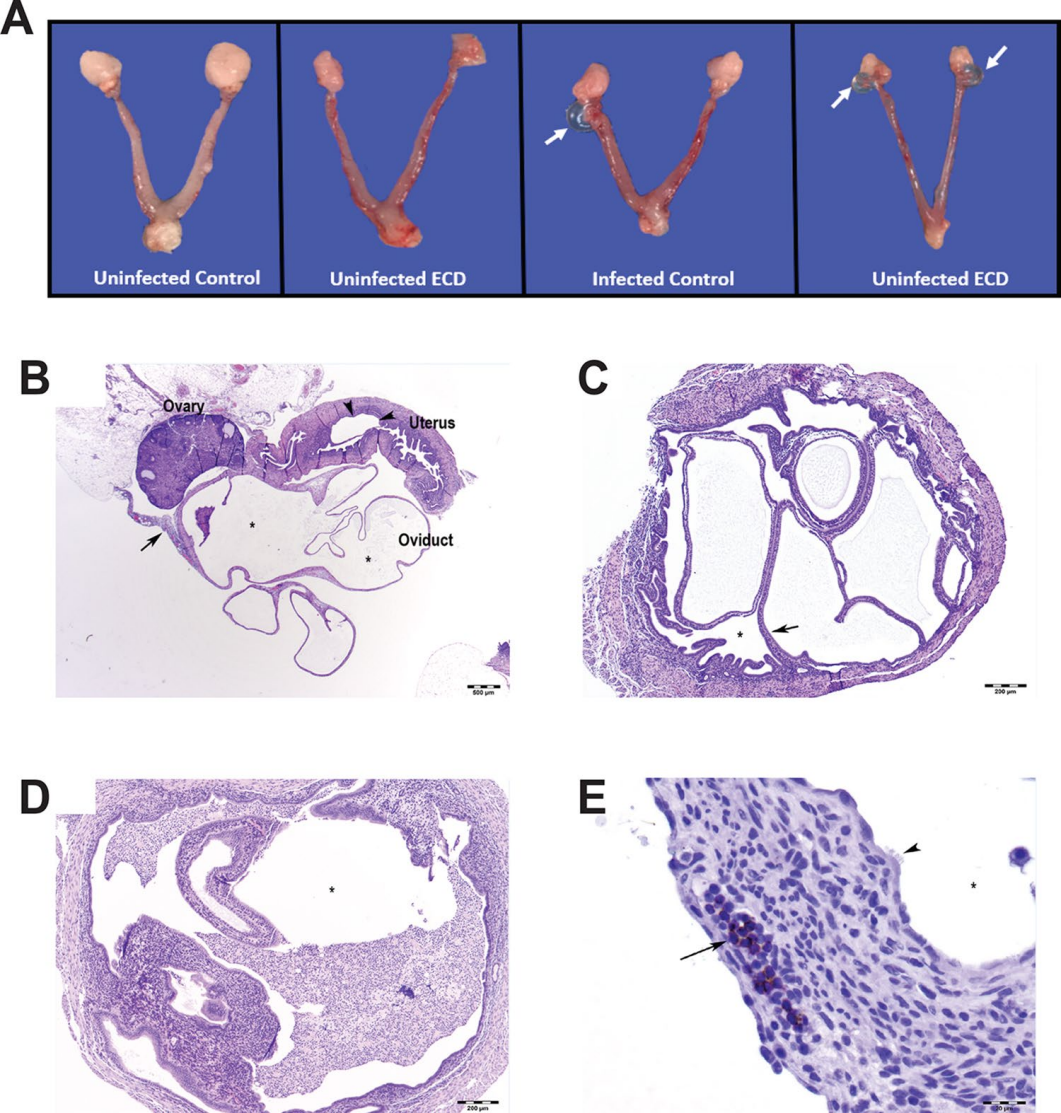
Effect of ECD on gross pathology and histopathology after *Chlamydia* infection (n = 6). Gross pathology. (**A**) Infected ECD mice had more periovarian cysts (white arrow) than control mice. (**B**) There was moderate periovarian and oviductal inflammation (long arrow), hydrosalpinx (asterisks), and cystic endometrial hyperplasia of the uterus (arrowheads). Hematoxylin and eosin (HE) stain. Bar = 500 µm. (**C**) Mouse uterus in infected ECD mice, with cystic endometrial hyperplasia (long arrow). Asterisk in uterus lumen. HE stain. Bar = 200 µm. (**D**) Mouse uterus in infected control mice, with cystic endometrial hyperplasia. Asterisk in uterus lumen containing large numbers of neutrophils. HE stain. Bar = 200 µm. (**E**) Mouse oviduct in infected ECD mice, with hydrosalpinx and lymphocytic (CD4 positive) inflammation (long arrow). Asterisk in dilated oviduct lumen (ampulla), lined by ciliated cells (arrowheads). Hematoxylin counterstain. Bar = 20 µm.

**Figure 4:**
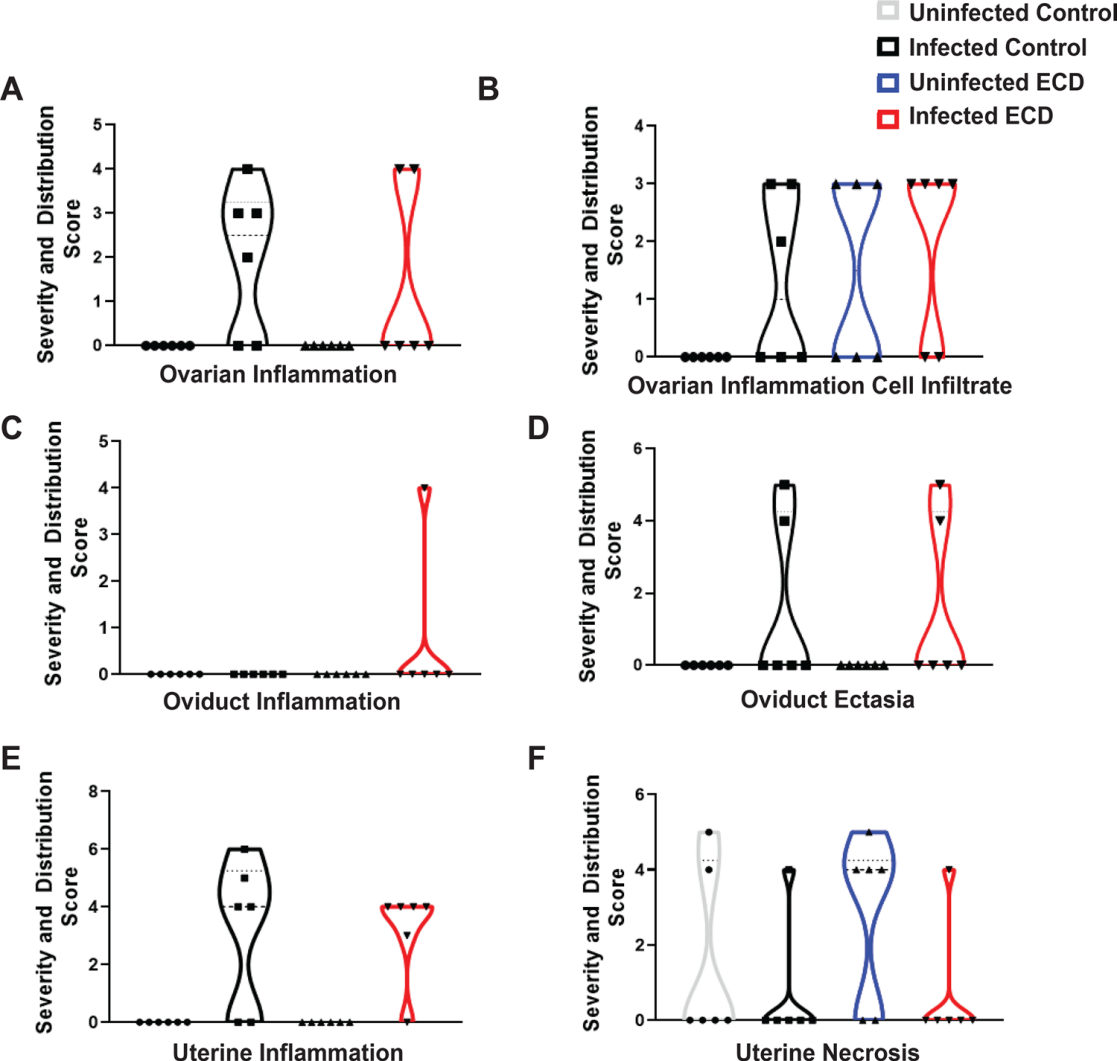
Severity and distribution of histopathology scores in ECD and control mice after *Chlamydia* infection. (**A**) Ovarian inflammation. (**B**) Ovarian inflammation cell infiltrate. (C) Oviduct inflammation. (**D**) Oviduct ectasia. (**E**) Uterine inflammation. (**F**) Uterine necrosis. Samples were collected 34 days post infection (n = 6).

The gross pathology of the genital tract of ZT3 infected ECD and control mice was determined (Fig. 3A). While there were no major differences in the incidence of gross lesions between ECD and control mice, there were differences at the cellular level with the histopathology showing that ECD mice had increased incidence of periovarian cysts or hydrosalpinx compared to control mice (Fig. 3A). Tissue alterations or lesions associated with chlamydial infection included lymphocytic and plasmacytic inflammation of the ovary (oophoritis), oviduct (salpingitis), and uterus (endometritis) as well as oviductal cysts (hydrosalpinx) and cystic endometrial hyperplasia (Fig. 3B–E). The lymphocytic inflammation contained increased numbers of CD4-position lymphocytes (Supplementary Table S1). The results indicate that tissue alterations or lesions associated with chlamydial infection were more accentuated in mice from the ECD groups. While control mice had similar lesions associated with chlamydial infection, their incidence and severity were lower. Ovarian inflammation was observed in infected ECD and infected control mice (Fig. 4A). Ovarian inflammation cell infiltrate was observed in infected and uninfected ECD and infected control mice (Fig. 4B). Oviduct inflammation was observed in infected ECD mice (Fig. 4C). ECD and infected control mice had oviduct ectasia and uterine inflammation, while uterine necrosis was observed in all groups (Fig. 4D–F). The incidence of microscopic findings is summarized in Supplementary Table S1, with the numbers of animals with lesions and lesion severity per group indicated. The microscopic findings for individual animals by group are presented in Supplementary Tables S2–S4. These results showed that the disruption in the circadian rhythms of the infected mice is associated with an increased incidence and severity in lesions within the genital tract.

### Effect of ECD on cytokine/chemokine production after Chlamydia infection

**Figure 5:**
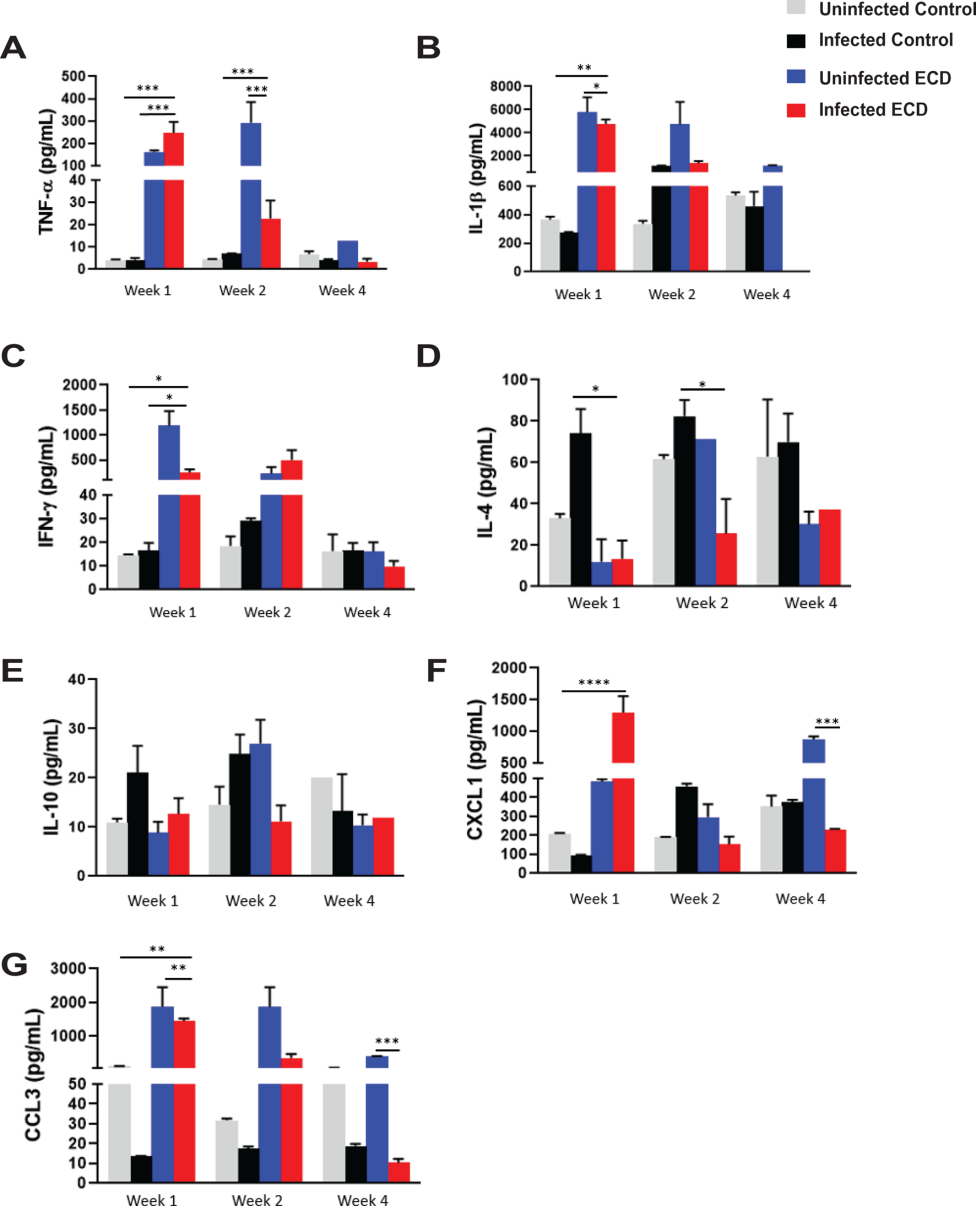
Effect of ECD on cytokine and chemokine secretion after *Chlamydia* infection. Cytokine and chemokine concentrations in vaginal lavages collected from ECD and control mice infected at ZT3 were determined (n = 12). (**A**) TNF-α. (**B**) IL-1β. (**C**) IFN-γ. (**D**) IL-4. (**E**) IL-10. (**F**) CXCL1. (**G**) CCL3. Uninfected and infected ECD and control mice were compared with each other. The data was analyzed using a one-way ANOVA and Tukey post hoc test. *p < 0.05; **p < 0.01; ***p < 0.001; ****p < 0.0001.

To determine the influence of ECD on the immune response generated following *C. muridarum* infection, we first determined the levels of cytokines and chemokines secreted in the vaginal lavage collected from mice infected at ZT3. One-week post infection, infected ECD mice had significantly higher levels of the proinflammatory cytokine TNF-α compared to uninfected ECD, infected and uninfected control mice (p < 0.001). In addition, the proinflammatory cytokine IL-1β was significantly higher in infected and uninfected ECD mice compared to infected and uninfected control mice (p < 0.001). At 2 weeks post infection, TNF-α and IL-1β levels were much lower compared to 1-week post infection (Fig. 5A,B). Infected and uninfected ECD mice had significantly higher secretion of the Th1 cytokine IFN-γ compared to infected control mice (p < 0.05) 1-week post infection (Fig. 5C). The Th2 cytokine IL-4 was significantly lower in uninfected and infected ECD mice compared to infected control mice 1- and 2-weeks post infection (p < 0.05) (Fig. 5D). There were no significant differences in IL-10 secretion at all timepoints post infection; however, the level of IL-10 was low all through for infected ECD mice (Fig. 5E). One-week post infection, infected ECD mice produced significantly higher levels of CXCL-1 and CCL3 compared to the other groups (p < 0.0001, p < 0.01 respectively), which then decreased 2 weeks post infection (Fig. 5F,G). The disruption in circadian rhythms in *Chlamydia* infected mice resulted in the dysregulated secretion of the cytokines and chemokines.

### Effect of ECD on anti-Chlamydia antibody production

**Figure 6:**
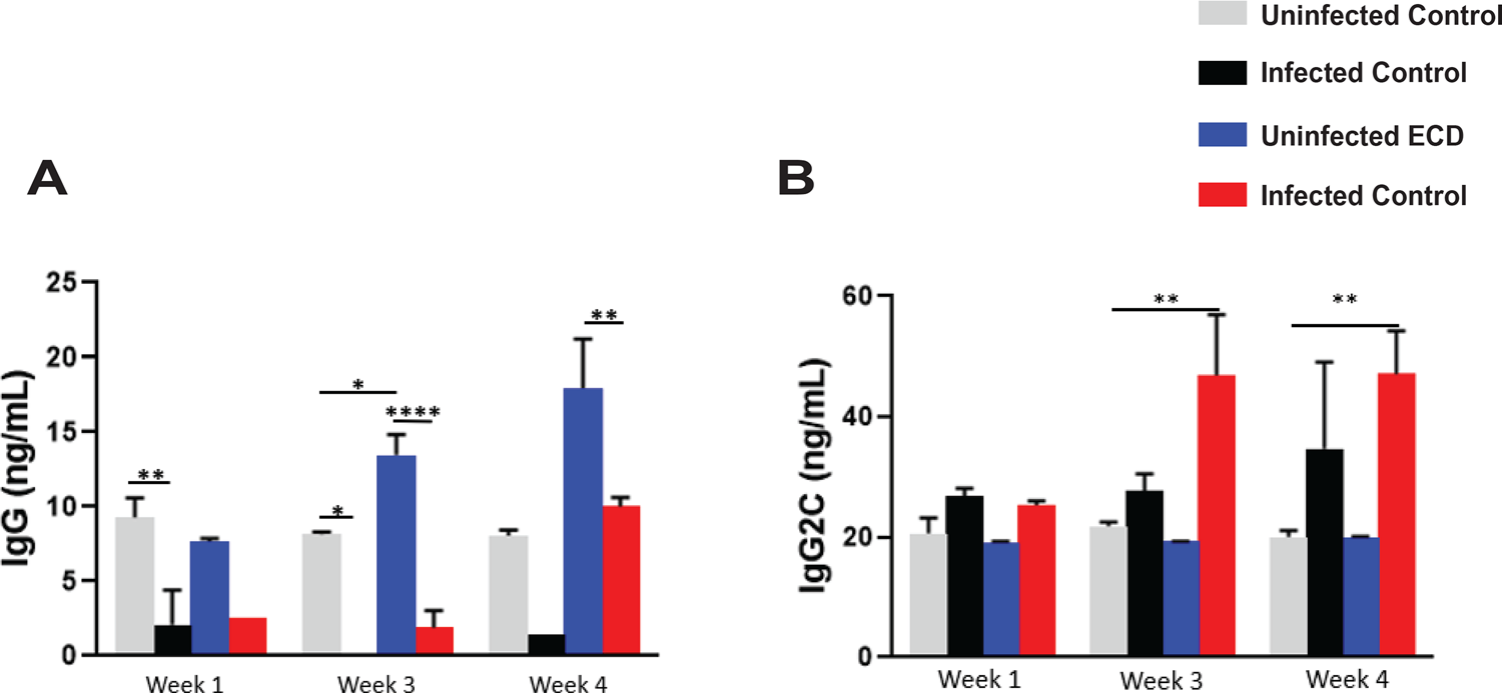
Effect of ECD on anti-chlamydial antibody secretion after *Chlamydia* infection. Anti-*Chlamydia* antibody concentrations were determined in vaginal lavages collected weekly from ECD and control mice (n = 12) infected with *C. muridarum* at ZT3. (**A**) IgG. (**B**) IgG_2C_. The data was analysed using a one-way ANOVA and Tukey post hoc test. **p < 0.01; ***p < 0.001; ****p < 0.0001.

To determine the effect of ECD on antibody production, we measured the amount of anti-chlamydial antibodies (IgG and IgG_2C_) secreted in vaginal lavage at the indicated timepoints after infection at the early rest period. The infected control mice elicited higher amounts of IgG antibodies compared to the infected ECD mice at all time points (Fig. 6A). However, the levels of IgG_2C_ were higher in the infected ECD mice compared to the infected control mice especially at 3- and 4-weeks post infection (p < 0.01) (Fig. 6B). These results imply that the disruption of circadian rhythms causes a change in the type of antibody response to chlamydial antigen.

### Effect of ECD on female mouse fertility after Chlamydia infection

**Figure 7:**
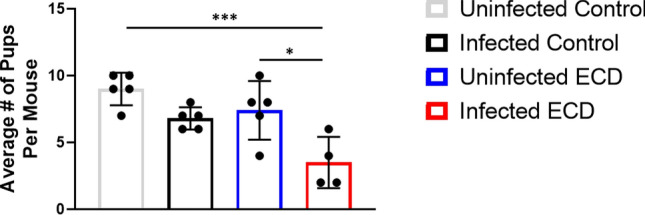
Effect of ECD on fertility after *Chlamydia* infection. Fertility of ECD and control mice were measured using a fertility assay after infection with *C. muridarum* at ZT3 (n = 6 per group). The fertility rate was determined by analyzing the number of pups per mouse. The data was analysed using a one-way ANOVA and Tukey post hoc test. *p < 0.05.

To assess the effect of ECD on the fertility rate of infected mice, the mean number of embryos in infected and uninfected ECD and control mice were compared. The results showed that the fertility rate of the infected control mice (with a fertility rate of 7.5) was significantly higher compared to the infected ECD mice (with a fertility rate of 3.5) (p < 0.05) (Fig. 7). This result suggests that the disruption of the circadian rhythms negatively impacts the fertility of *Chlamydia* infected mice.

### Effect of ECD on Chlamydia infection during the early active period

**Figure 8:**
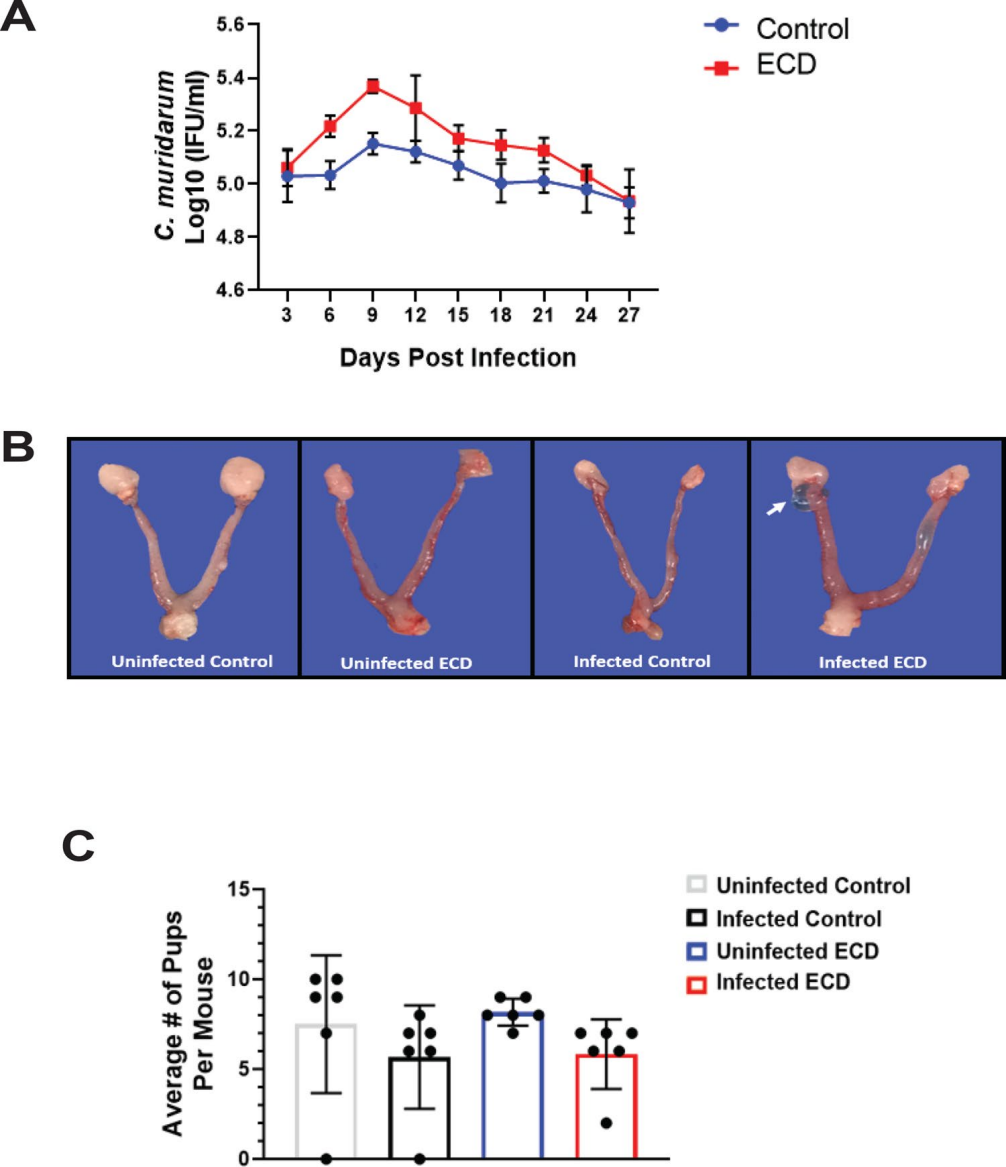
Effect of ECD on chlamydial pathogenesis after *Chlamydia* infection in the early active period. (**A**) *Chlamydia* infectivity in ECD and control mice (n = 12 per group) infected at ZT15 was determined. Data was analyzed using a two-way repeat measure ANOVA and Tukey post hoc test. (**B**) ECD mice had periovarian cysts (next to the ovary, as indicated by the white arrow), while infected control mice did not have periovarian cysts (n = 6 per group). (**C**) Fertility of ECD and control mice infected with *C. muridarum* at ZT15 was determined by analyzing the number of pups per mouse (n = 6 per group). The data was analysed using a one-way ANOVA and Tukey post hoc test.

We had previously reported that time of day of infection plays a role in chlamydial pathogenesis and disease outcomes. In this study, we determined if infection at ZT15 (early active period), would mitigate the effect of ECD on the outcomes observed in mice that were infected at ZT 3 (early rest period; Figs. 2, 3, 4, 5, 6, 7). *C. muridarum* infectivity results showed that following infection at ZT15, infected ECD mice had moderately higher but not significant *Chlamydia* loads compared to control mice (Fig. 8A). Furthermore, although hydrosalpinx was observed in infected ECD mice, limited tubal pathologies were associated with ECD mice infected at ZT15 (Fig. 8B), indicating that the consequences of chlamydial infection were less accentuated in ECD mice infected at ZT15. The incidence and severity of lesions associated with chlamydial infection were minimal to absent in control mice infected at ZT15. Tissue alterations or lesions associated with chlamydial infection included oophoritis, salpingitis, endometritis, hydrosalpinx and cystic endometrial hyperplasia. The lymphocytic inflammation contains increased numbers of CD4-positive lymphocytes (Supplementary Table S1). Ovarian inflammation was observed in infected ECD mice (Supplementary Fig. S1A). Ovarian inflammation cell infiltrate was observed in uninfected and infected ECD mice and infected control mice (Supplementary Fig. S1B). Oviduct degeneration was only observed in infected ECD mice (Supplementary Fig. S1C). Oviduct hyperplasia was observed in infected ECD and infected control mice (Supplementary Fig. S1D). Uterine endometrial hyperplasia was observed in infected ECD mice (Supplementary Fig. S1E). Uterine necrosis was observed in all ECD and control groups (Supplementary Fig. S1F). The incidence of microscopic findings associated with ZT15 infection is summarized on Supplementary Table S1, in which the numbers of animals with lesions and lesion severity per group are indicated. The microscopic findings for individual animals by group are presented in Supplementary Tables S2–S4. These results show that the disruption in the circadian rhythms of the infected mice is associated with moderate increase in incidence and severity in genital tract pathology. Infected ECD mice had significantly higher secretion of the proinflammatory cytokines TNF-α and IL-1β compared to infected control mice. The Th1 cytokine IFN-γ was higher in the infected ECD mice (p < 0.0001) (Supplementary Fig. S2A–C). IL-4 levels were higher in infected control mice compared to infected ECD mice, the IL-4 levels of the infected ECD mice were higher than that of ECD mice infected at ZT3 (Supplementary Fig. S2D). There were no significant differences in IL-10 levels, the IL-10 levels were higher than that secreted after infection at ZT3 (Supplementary Fig. S2E). Secretions of the chemokines CXCL1 and CCL3 were significantly higher in infected ECD mice compared to infected control mice one-week post infection (p < 0.0001) (Supplementary Fig. S3A,B), there was a decrease in the chemokine levels by 2 weeks post infection, which is like the trend observed for mice infected at ZT3. Antibody levels were measured to determine the effect of ECD on the protection from *C. muridarum* infection. The infected ECD mice had significantly less IgG than the uninfected control mice at all time points (p < 0.01, p < 0.01, p < 0.0001) (Supplementary Fig. S4A). The infected ECD mice had significantly higher IgG_2C_ than infected control mice (p < 0.01) (Supplementary Fig. S4B).

Fertility assay showed that in mice infected at ZT15, the fertility rate was not significantly higher in infected control mice compared to the infected ECD mice (Fig. 8C). This was different from what was observed after infection at ZT3, with the number of pups in the ZT15 infected ECD mice almost doubled that of ECD mice infected at ZT3. The trend in fertility was identical for mice infected at ZT3 and ZT15; however, the effect of ECD appears milder in mice infected at ZT15 corroborating our earlier work where we showed that the time of day of infection was important in determining the extent of chlamydial pathogenesis.

## Discussion

We had previously reported a role for time of day of infection in the pathogenesis of *Chlamydia* infection^13^. We reported that *Chlamydia* infectivity and pathogenesis in mice increased when mice were infected during the early rest period (ZT3) compared to the early active period (ZT15). This led us to postulate that circadian rhythms might be important in determining which individuals go on to have disease and its associated complications. In the current study, we utilize a mild ECD model used in several recent studies^14–16^. The ECD model simulates shift work in mice, by disrupting their circadian rhythms through the advancing of the light schedule^17,18^. Shift work in humans occurs when individuals work at abnormal times and thus have more exposure to light, and are exposed to light during their biological night, than unlike people who work from 9:00 am to 5:00 pm^14–16^. The effect of shift work on health outcomes is profound. Shift work has been reported to be associated with several disease conditions such as cardiovascular disease, diabetes, high blood pressure, obesity, and breast cancer^1,3–5,19^. The possibility of reducing the number of people involved in shift work is very slim, since growing economies need individuals to work around the clock to produce the goods and services required for an ever-increasing middle class^19^. Therefore, there is a need to understand the role of this phenomena in health outcomes. It should be noted that very little is known about the effect of shift work on infectious disease in general and sexually transmitted infections. In this study, we report the effect of shift work in chlamydial pathogenesis.

We observed that ECD mice infected during the early rest period, ZT3, had pronounced pathological outcomes^13^ and had significant but moderately higher chlamydial burden from days 12 to 24 than infected control mice housed in a standard light cycle. This indicates that mice that have had their circadian rhythms disrupted had higher *Chlamydia* loads after infection, suggesting that genes/proteins in host cells responsible for controlling infection might be dysregulated. Infected ECD mice had more ovarian cysts than infected control mice, however this was not statistically significant. The mice were infected once, and we did not notice the more robust pathological outcome seen after two or more infections. Additionally, mice were infected at 9 weeks old, due to the time it takes to perform the ECD model, so they were infected at an older age. Older mice are more resistant to chlamydial infection. Histopathology results showed that tissue alterations associated with chlamydial infection were predominant in ECD mice compared to control mice^20^. The similarity in uterine necrosis noticed in all mice groups could be due to histological and functional remodeling of the endometrium during the estrous cycle^21,22^. The lymphocytic inflammation contained increased numbers of CD4-positive lymphocytes, which are associated with *Chalmydia*l^23–26^. In this study we housed the ECD mice under normal LD conditions after infection. We speculate that the infectivity and pathological outcomes would have been worse if we had continued the disruption of the circadian rhythms during the infection period.

Pathology from chlamydial infection is associated with host immune response to *Chalmydia*^20,23,27^. Cytokines and chemokines are important mediators of this immune response^28–30^. Infected ECD mice had higher secretions of the proinflammatory cytokines TNF-α and IL-1β in the first week of infection tapering off by the second week^15,31^. Excessive secretion of proinflammatory cytokines is associated with adverse pathological outcomes in the genital tract^32–35^. The high levels of proinflammatory cytokines in the first week of infection is indicative of the pathological damage observed in ECD mice. Infected and uninfected ECD mice had higher levels of IFN-γ, and lower levels of IL-4 compared to infected and uninfected control mice. The protective immune response against *Chlamydia* is a Th1 response rather than Th2 response^36^. The high levels of IFN-γ in ECD mice might be the cause of the adverse pathological outcome in these mice. The proinflammatory properties of IFN-γ appears additive to that of TNF-α and IL-1β. Infected control mice had significantly higher levels of the Th2 anti-inflammatory cytokine IL-4^37^, which can dampen the inflammatory properties of TNF-α and IL-1β. In the ECD mice this proinflammatory activity are not being inhibited. The levels of IL-10, responsible for controlling and balancing the effects^38–40^, in infected ECD mice was low throughout the infection period, thus playing a minimal role in mitigating the effect of the proinflammatory cytokines.

Neutrophil recruitment to the site of *Chlamydia* infection is associated with pathological outcomes such as scarring, swelling and edema^44–47^. CXCL1 and CCL3 are responsible for recruiting neutrophils and macrophages to the site of infection^41–44^. The high secretion of CXCL1 and CCL3 in ECD mice leads to the recruitment of more innate immune cells to the site of the infection increasing the possibility of having adverse pathological outcomes. It has been reported that ECD disrupts immune response, our results corroborate that study but with emphasis on *Chlamydia* infection^16^. The circadian clock has been reported to be important in immune cells function^13,45–49^.

Immune response to chlamydial infection involves the production of antibodies which provides an indication of the amount of chlamydial antigen present or the extent of protection^50–52^. We analyzed the amounts of IgG and IgG_2C_ secreted in the vaginal milieu. The results showed that infected control mice had higher IgG and lower IgG_2C_ than infected ECD mice in the third and fourth week of infection. The role of antibodies in the immune response against *Chlamydia* is not fully understood with arguments being made that antibodies are not important in *Chlamydia* clearance^53–57^. In infected control mice IgG is predominant, while in infected ECD mice IgG_2C_ is predominant, this could be associated with the high load of *Chlamydia* in ECD mice, compared to infected control mice. The dominant immunoglobulin isotype in the genital tract is IgG^58^. IgG2C is the predominant anti-chlamydial IgG antibody in C57BL/6 Mice^25,59^. Mouse IgG2c preferentially bind protein antigens, activate complement, and function as opsonins^60,61^. Anti-*Chlamydia* IgA responses, in both mice and humans, are of shorter duration, more variable, compared with IgG responses^62,63^. IgG2c has also been linked to a Th1 response in *Chlamydia* infection^60,64^. This suggests that antibodies might be playing a role in the immune response against *Chlamydia* and not just being an indicator of the bacterial burden.

To further understand the effect of ECD on chlamydial pathogenesis, we performed fertility assay. There was a difference in fertility rate, number of pups per mouse, between ECD and control mice and this difference was exacerbated in mice infected with *Chlamydia*. This shows that ECD had a significant effect on mice fertility rate and that *Chlamydia* infection made this outcome worse. We do not fully understand how the disruption of the circadian rhythms is leading to this outcome, but we know that *Chlamydia* infection causes scaring and fibrosis within the genital tract that can lead to tubal blockage^10,65,66^, it appears that this process is accelerated in ECD mice.

We previously reported that the time of day of infection played a role in determining chlamydial pathogenesis^13^. Based on this premise, we determined the effect of ECD on mice infected at the early active period (ZT15; associated with milder *Chlamydia* infectivity and disease outcomes). The results showed that the effect of ECD is ameliorated by infection at ZT15, with reduced infectivity and pathology and better fertility rates in ECD mice infected at ZT15 compared with ECD mice infected at ZT3. These results appear to tally with our previous study and suggests that the time of day of infection has role to play in ECD mice^9,13,67^.

This study shows for the first time an association between the disruption of circadian rhythms, in this case through ECD, with the complications associated with chlamydial infection. Individuals performing shift work have a higher likelihood developing certain disease conditions compared with individuals working normal work schedules. The results from this study suggests that we can add diseases associated with chlamydial infection to those conditions. It shows that shift work is a factor that should be analyzed when studying the effect of *Chlamydia* infection within the population, shift workers might have more enhanced pathological disease. Knowing the time of day of chlamydial infection is also important, as infection in the early active period is associated with less disease complications. This is important in figuring out a way to reduce the outcomes of chlamydial pathogenesis in shift workers.

## Methods

### Animals

#### Normal light: dark cycle

Female, C57BL/6J mice (Jackson Laboratory, Bar Harbor, MA) at 5 weeks old were housed in cages under normal light: dark cycle (LD) conditions of 12 h lights on and 12 h lights out for 4 weeks (Control) before infection (Fig. 1). The mice were further kept under LD conditions of 12 h lights on and 12 h lights out for another 4 weeks during the infection period.

#### Environmental circadian disruption (ECD) model

Female, C57BL/6 J mice (Jackson Laboratory, Bar Harbor, MA) at 5 weeks old were housed in cages and received 6-h advance in light cycle every week for four weeks before infection with 1 × 10^6^ *C. muridarum* (S Fig. 1)*.* The mice were then placed back onto LD 12:12 during the infection period as previously described^16,17^.

### Chlamydia stock

*Chlamydia muridarum niggs* (*C. muridarum*) stocks (Centers for Disease Control and Prevention, Atlanta, GA) were diluted in sterile Sucrose Phosphate Glutamate (SPG) transport media to a final concentration of 1 × 10^6^ Infectious Units (IFUs).

### *C. muridarum* propagation

*Chlamydia* stock was grown in flask containing McCoy cells for 3 h. Cycloheximide was added to the flasks and incubated at 37 °C for 24 h. The cell monolayer was scraped and pooled in a 50 ml conical tube and sonicated for 60 s then centrifuged at 2000 rpm for 10 min a 4 °C. The supernatant was removed and centrifuged at 16,000 rpm for 30 min at 4 °C to pellet the *Chlamydia* elementary bodies. The pellet was resuspended in sucrose phosphate glutamate (SPG) buffer. Aliquots were stored at − 80 °C.

### Infectivity assay

All ECD and control mice were subcutaneously injected with 2.5 mg/ml Depo Provera, medroxyprogesterone acetate (Pfizer, New York, NY) in sterile Phosphate Buffer Saline (PBS) to synchronize the estrous cycle. Mice were intravaginally infected seven days later (while under isoflurane anesthesia), with sham infection (20 ul SPG) or 1 × 10^6^ *C. muridarum* at 10:00 am (Zeitgeber Time (ZT) 3, early rest period, n = 12) or 10:00 pm (ZT15, early active period, n = 12), which was three hours after the lights were turned on or off in the room (7:00 am lights on (ZT 0), 7:00 pm lights off (ZT12). *Chlamydia* infected mice were swabbed every three days for 27 days and the *Chlamydia* was isolated and cultured to track the progression and clearance of the infection.

### *C. muridarum* isolation from vaginal swabs

McCoy cells were plated at a concentration of 1 × 10^5^ cells per well in a 24 well plate. Conical tubes containing vaginal swabs and glass beads in SPG buffer were vortexed on ice for 30 s. Swabs were removed and contents of conical tubes were sonicated for 20 s on ice then vortexed a second time for 10 s. 300 µl of inoculum was transferred into each well. The plates were centrifuged for 1 h at 2,200 rpm, then incubated at 37 °C for 1 h. The supernatant was removed and 1 ml of warm Iscove media (with no antibiotics) plus 10% FBS and 1.5 mg/ml cycloheximide and 15% FBS was added and the plate was incubated for 48 h at 37 °C.

### *C. muridarum* staining

Media was removed from 24 well plates and washed two times with PBS. Cells were fixed with 1 ml ice cold 100% methanol for 1 h. Methanol was removed and one drop of *Chlamydia* Pathfinder (Bio-Rad Laboratories Inc., Hercules, CA, USA) was added and the plate was incubated for 1 h in the dark. The *Chlamydia* Pathfinder was removed, and the plates were washed with distilled water two times. The *Chlamydia* inclusions in the plates were counted using a fluorescence microscope.

### Pathology

Euthanasia was performed using carbon dioxide and cervical dislocation between 10:00 am and 1:00 pm. Mice were immediately dissected, and the reproductive tract harvested and preserved in 10% Neutral Buffer Formalin (Thermo Fisher Scientific, Newark, DE, USA) and stored at 4 °C. Following euthanasia, the number of periovarian cysts and tubal dilations were independently assessed by 2 persons. Tissues were trimmed, routinely processed, embedded in paraffin, cut approximately into 5 µm sections, and stained with hematoxylin and eosin. Histopathological examination consisted of evaluation of the tissues for the presence or absence of inflammation, hydrosalpinx, endometrial hyperplasia (simple, papillary, cystic), uterine ectasia, degeneration, (apoptotic) necrosis, myxedema, adenomyosis, and fibroplasia. Histopathologic severity scores were assigned as grades 0 (no significant histopathological alterations); 1 (minimal); 2 (mild); 3 (moderate); or 4 (severe) based on increasing extent and/or complexity of change, unless otherwise specified. Lesion distribution was recorded as focal, multifocal, or diffuse, with distribution scores of 1, 2, or 3, respectively^13^. The histopathology was performed by a qualified pathologist.

### Immunohistochemistry

Immunohistochemistry was performed on an automated Stainer (Nemesis 3600, Biocare Medical, Concord, CA). A rat monoclonal antibody for CD4 (Thermo Fisher Scientific, 13-9766-82) at a dilution of 1:100 for 60 min was used. Antigen retrieval on tissue sections was achieved using Citrate Solution 10X (BioGenex, Fremont, CA) at a dilution of 1:10 for 15 min at 110 °C. A biotinylated, rabbit anti-rat antibody at a dilution of 1:100 (Vector Laboratories, Burlingame, CA) was utilized to detect the target, and immunoreaction was visualized using 3,3-diaminobenzidine (DAB, Biocare Medical, Concord, CA) substrate for 12 min counterstained with hematoxylin^68^. Spleen tissue from a mouse was used as positive control.

### Vaginal lavage

Vaginal lavage (wash) was performed once a week for four weeks following infection. Mice had pipette tip containing 60 µl sterile PBS inserted into the vagina. The PBS was dispensed and drawn up 10 times then transferred into an Eppendorf tube. The lavage was immediately placed on dry ice following collection and stored at − 80 °C. All washes were collected between 10:00 am and 12:00 pm.

### Cytokine and chemokine assay

The concentration of cytokines and chemokines (Tumor Necrosis Factor [TNF]-α, Interleukin [IL]-1β, Interferon [IFN]-γ, IL-10, IL-4, C-X-C motif ligand [CXCL]-1, and C-C motif chemokine ligand [CCL]-3) in the vaginal lavage were determined using the R&D Systems Magnetic Luminex Assay, Mouse Premixed Multi-Analyte Kit (R&D, Hercules, CA) in accordance with the manufacturer's protocol^13^. The concentration of cytokine/chemokine in each sample was obtained by extrapolation from a standard calibration curve. The mean and SD for replicate samples were calculated.

### Enzyme linked immunosorbent assay

Determination of concentrations of *Chlamydia* specific antibody isotypes, Immunoglobulin (Ig) IgG and IgG_2c_, in vaginal lavage of ECD or control mice were measured by standard ELISA procedure described previously^13,69^. Briefly, 96-well microtiter plates (Nunc Life Technologies, Rochester, NY) were coated with 20 µg/ml of chlamydial antigen (i.e., UV-inactivated *C. muridarum* elementary bodies) in 50 µl of PBS at 4 °C overnight. Plates were blocked for 1 h with 1% bovine serum albumin containing 5% goat serum in PBS/T and 50 µl of vaginal lavage in two-fold serial dilutions was added per well. Plates were incubated with 50 µl of horseradish peroxidase-conjugated goat anti-mouse IgG isotypes (Southern Biotechnology Associates, Inc., Birmingham, AL, USA.) for 1 h and developed with 2,2′-azino-bis (3-ethylbenzthiazoline-6-sulfonic acid) (ABTS). The optical density was measured at 490 nm on a microplate reader. Results, generated simultaneously with a standard curve, display data sets corresponding to absorbance values as mean concentrations (ng/ml) ± SD and represent the mean values from triplicate experiments.

### Fertility assay

Five weeks after infection, infected (ZT3 and ZT15) and uninfected female ECD mice and their controls were placed in cages with proven fertile male C57BL/6J mice (Jackson Laboratory, Bar Harbor, MA), at two females to one male mouse. The female mice were weighed every three days after 1 week until they have gained approximately 10 g to confirm pregnancy. Once pregnancy has been confirmed, mice were sacrificed and dissected to determine the number of pups per mouse^70,71^.

### Statistical analysis

One-way analysis of variance (ANOVA) was used to analyze the statistical differences in immune response and fertility rate between infected and non-infected ECD mice and their controls. Two-way repeat measure ANOVA was used to determine the difference in infectivity between the treatment groups. In addition, we also did a Tukey post hoc test (for multiple comparisons by comparing the mean of each group to the mean of every other group) after the one or two-way ANOVA, to determine the actual statistical relationship between the treatment groups. Statistical significance was determined at P < 0.05. GraphPad Prism (La Jolla, CA) was used for analyzing the data.

### Animal protocol approval statement

This study was carried out in strict adherence to the recommendations in the Guide for the Care and Use of Laboratory Animals of the National Institutes of Health. The study protocol was approved by the Institutional Animal Care and Use Committee (IACUC) of Morehouse School of Medicine (Protocol Number: 16–24).

### Ethics approval and consent to participate

The Institutional Animal Care and Use Committee (IACUC) of Morehouse School of Medicine approved the study protocol (Protocol Number: 16–24).

### Consent for publication

Not applicable.

## Supplementary information

Supplementary Table S1.

Supplementary Table S2.

Supplementary Table S3.

Supplementary Table S4.

Supplementary Figures

## Data Availability

All data and results have been added to this manuscript and the supplementary material section.
