# Author Correction: Shift work influences the outcomes of *Chlamydia* infection and pathogenesis

**DOI:** 10.1038/s41598-020-79099-z

**Published:** 2020-12-11

**Authors:** Stephanie R. Lundy, Shakyra Richardson, Anne Ramsey, Debra Ellerson, Yan Fengxia, Sunny Onyeabor, Ward Kirlin, Winston Thompson, Carolyn M. Black, Jason P. DeBruyne, Alec J. Davidson, Lilly C. Immergluck, Uriel Blas-Machado, Francis O. Eko, Joseph U. Igietseme, Qing He, Yusuf O. Omosun

**Affiliations:** 1 Department of Microbiology, Biochemistry & Immunology, Morehouse School of Medicine, 720 Westview Drive, S.W., Atlanta, GA 30310 USA; 2 Department of Neurobiology, Morehouse School of Medicine, Atlanta, GA 30310 USA; 3 Centers for Disease Control & Prevention (CDC), Atlanta, GA 30333 USA; 4 Department of Community Health and Preventive Medicine, Morehouse School of Medicine, Atlanta, GA 30310 USA; 5 Department of Pharmacology, Morehouse School of Medicine, Atlanta, GA 30310 USA; 6 Department of Physiology, Morehouse School of Medicine, Atlanta, GA 30310 USA; 7 Pediatric Clinical & Translational Research Unit, Clinical Research Center, Morehouse School of Medicine, Atlanta, GA 30310 USA; 8 Athens Veterinary Diagnostic Laboratory, Department of Pathology, College of Veterinary Medicine, University of Georgia, Athens, GA 30602 USA

Correction to: *Scientific Reports* https://doi.org/10.1038/s41598-020-72409-5, published online 21 September 2020

This Article contains an error in Figure 3, where the last image in panel (a) is incorrectly labelled ‘Uninfected ECD’. The correct Figure 3 appears below as Figure 1.

**Figure 1:**
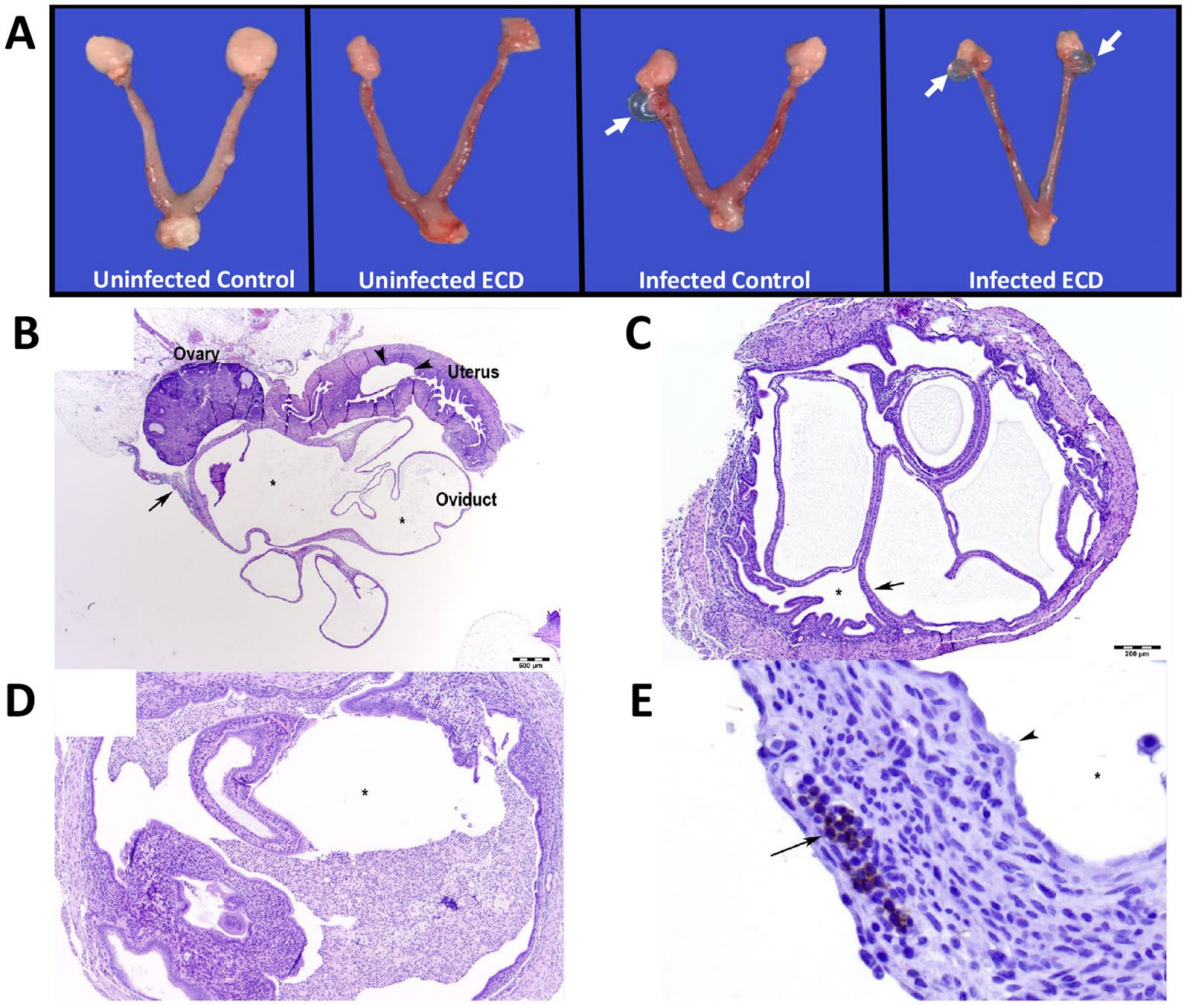
A correct version of the original Figure 3.

