# Supplementary material for: Shift work influences the outcomes of Chlamydia infection and pathogenesis
Source: Sci Rep. 2020 Sep 21;10:15389. doi: 10.1038/s41598-020-72409-5 (PMC7505842; doi:10.1038/s41598-020-72409-5)
Supplement: Supplementary file 5 — Supplementary Figures [file 41598_2020_72409_MOESM5_ESM.docx]

**Supplementary data**

**Shift Work Influences the Outcomes of Chlamydia Infection and Pathogenesis**

Stephanie R. Lundy^1^, Shakyra Richardson^1^, Anne Ramsey^2^, Debra Ellerson^3^, Yan Fengxia^4^, Sunny Onyeabor^4^, Ward Kirlin^5^, Winston Thompson^6^, Carolyn M. Black^3^, Jason P. DeBruyne^5^, Alec J. Davidson^2^, Lilly C. Immergluck^1,7^, Uriel Blas-Machado^8^, Francis O. Eko^1^, Joseph U. Igietseme^1,3^, Qing He^1,3, 9^ and Yusuf O. Omosun^1, 3^.

^1^Department of Microbiology, Biochemistry & Immunology, Morehouse School of Medicine, Atlanta, GA 30310 USA; ^2^Department of Neurobiology, Morehouse School of Medicine, Atlanta, GA 30310 USA; ^3^Centers for Disease Control & Prevention (CDC) Atlanta, GA 30333 USA; ^4^Department of Community Health and Preventive Medicine, Morehouse School of Medicine, Atlanta, GA 30310 USA; ^5^Department of Pharmacology, Morehouse School of Medicine, Atlanta, GA 30310 USA;  ; ^6^Department of Physiology, Morehouse School of Medicine, Atlanta, GA 30310 USA; ^7^Pediatric Clinical & Translational Research Unit, Clinical Research Center, Morehouse School of Medicine, Atlanta, GA 30310 USA; ^8^Athens Veterinary Diagnostic Laboratory, Department of Pathology, College of Veterinary Medicine, University of Georgia, Athens, GA 30602 USA .

^9^ Deceased.


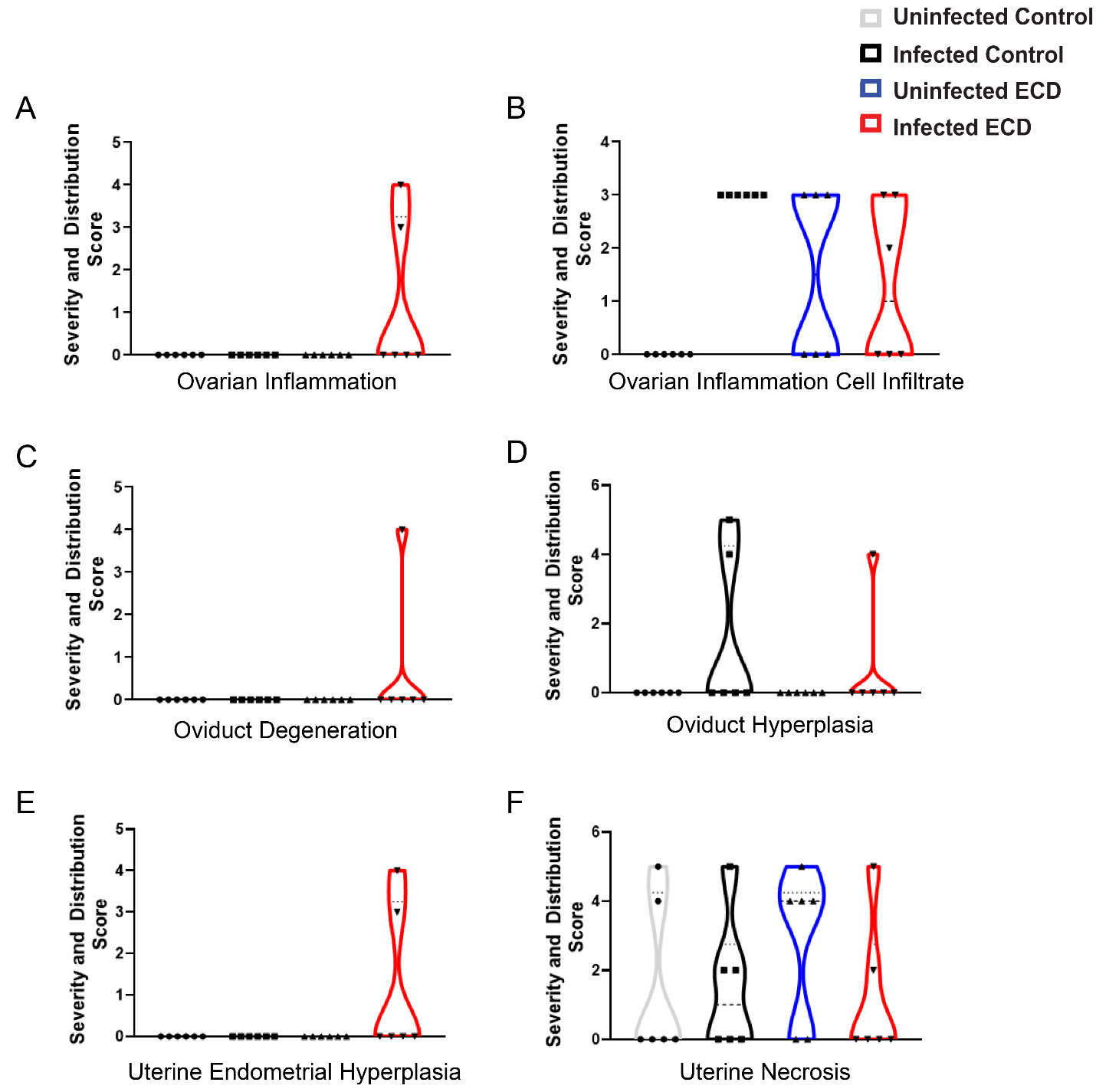


S Figure 1: Severity and distribution of histopathology scores in ECD and control mice infected with *C. muridarum* during the early active period. A) Ovarian inflammation. B) Ovarian inflammation infiltrate. C) Oviduct degeneration. D) Oviduct hyperplasia. E) Uterine endometrial hyperplasia. F) Uterine necrosis.


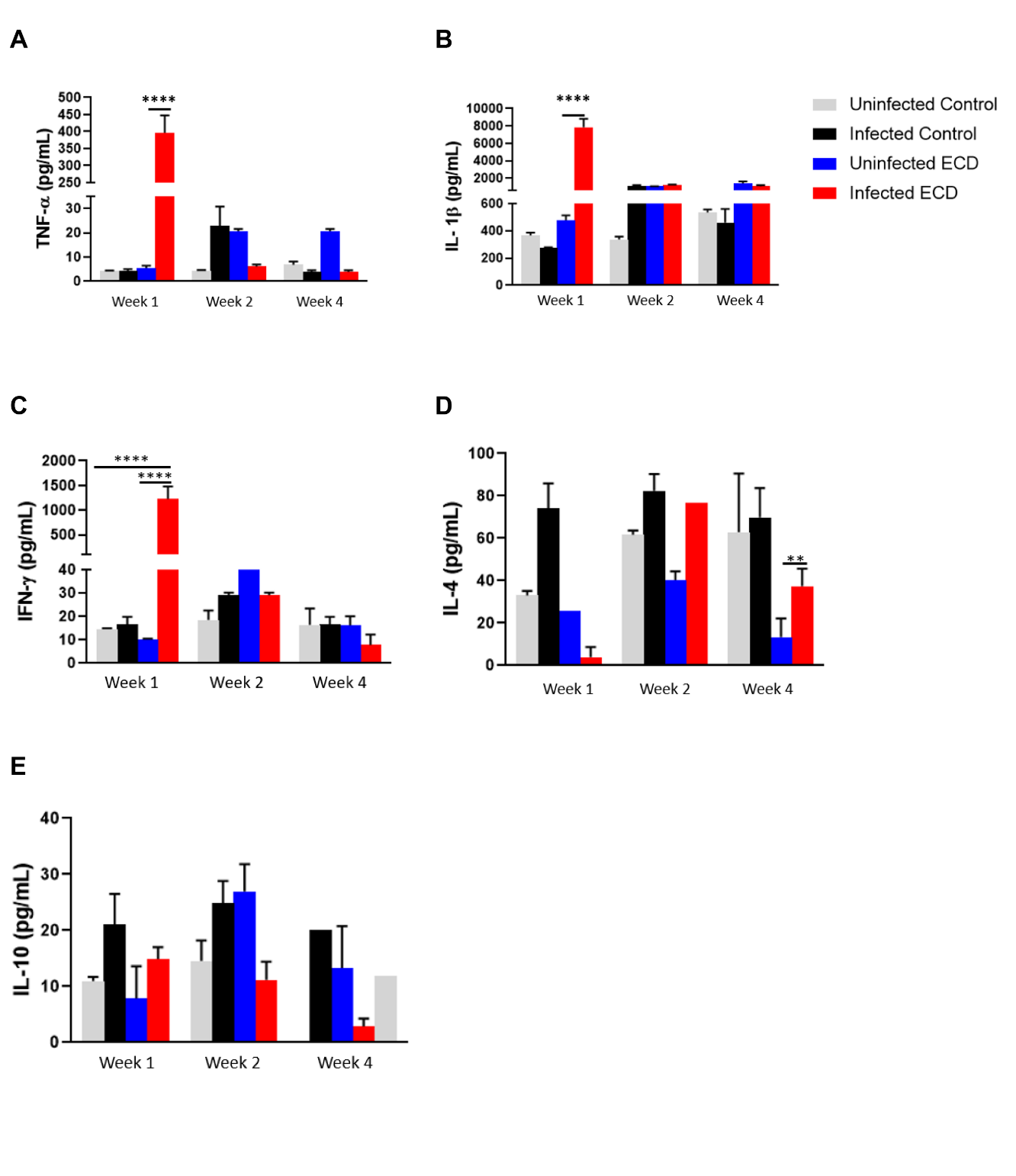
S Figure 2: Effect of ECD on cytokine secretion during the early active period. Cytokine concentrations were determined in vaginal lavage collected from ECD and control mice (n=12) infected with C. muridarum at ZT15. A) TNF-α. B) IL-1β. C) IFN-γ. D) IL-4 secretions. E) IL-10. The data was analysed using a one-way ANOVA and Tukey post hoc test. * p<0.05; **p<0.01; ***p<0.001; ****p<0.0001.

*
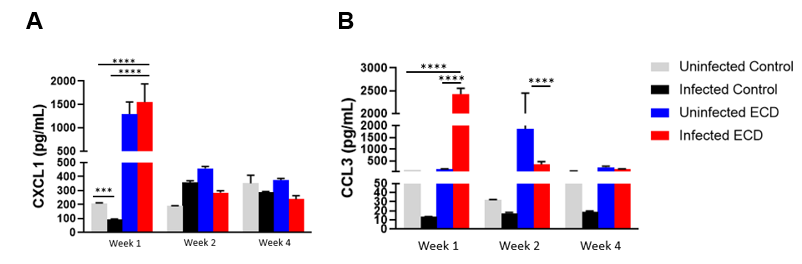
*

S Figure 3: Effect of ECD on chemokine secretion during the early active period. Chemokine concentrations were determined in vaginal lavage collected from ECD and control mice (n=12) infected with C. muridarum at ZT15. A) CXCL1. B) CCL3. The data was analysed using a one-way ANOVA and Tukey post hoc test. * p<0.05; **p<0.01; ***p<0.001; ****p<0.0001.

‘


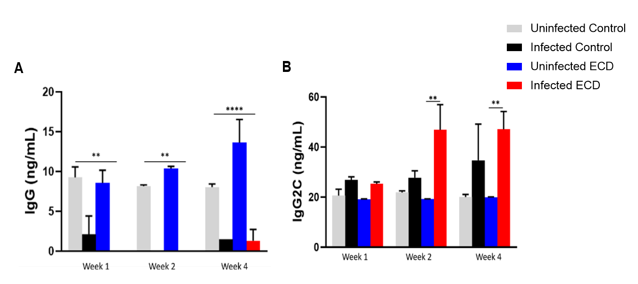


S Figure 4: Effect of ECD on anti-chlamydial antibody secretion during the early active period. Anti-chlamydia antibody concentrations were determined in vaginal lavages collected weekly from ECD and control mice (n=12) infected with *C. muridarum* at ZT15. A) IgG. B) IgG2C. The data was analysed using a one-way ANOVA and post hoc test. * p<0.05; **p<0.01; ***p<0.001; ****p<0.0001.
